# Laser Acupuncture for Postoperative Pain Management in Cats

**DOI:** 10.1155/2015/653270

**Published:** 2015-06-11

**Authors:** Virgínia I. Marques, Renata N. Cassu, Felipe F. Nascimento, Rafaela C. P. Tavares, Giulliane C. Crociolli, Rafael C. Guilhen, Gabriel M. Nicácio

**Affiliations:** ^1^Postgraduate Program in Animal Science, Oeste Paulista University, 19067-175 Presidente Prudente, SP, Brazil; ^2^Faculty of Veterinary Medicine, Oeste Paulista University, 19067-175 Presidente Prudente, SP, Brazil

## Abstract

The aim of this study was to evaluate laser acupuncture as an adjuvant for postoperative pain management in cats. Twenty cats, undergoing ovariohysterectomy, were sedated with intramuscular (IM) ketamine (5 mg kg^−1^), midazolam (0.5 mg kg^−1^), and tramadol (2 mg kg^−1^). Prior to induction of anaesthesia, the subjects were randomly distributed into two groups of 10 cats: Laser: bilateral stomach 36 and spleen 6 acupoints were stimulated with infrared laser; Control: no acupuncture was applied. Anaesthesia was induced using intravenous propofol (4 mg kg^−1^) and maintained with isoflurane. Postoperative analgesia was evaluated by a blinded assessor for 24 h following extubation using the Dynamic Interactive Visual Analogue Scale and Multidimensional Composite Pain Scale. Rescue analgesia was provided with IM tramadol (2 mg kg^−1^), and the pain scores were reassessed 30 min after the rescue intervention. If the analgesia remained insufficient, meloxicam (0.2 mg kg^−1^ IM, single dose) was administered. Data were analyzed using *t*-tests, the Mann-Whitney test, and the Friedman test (*P* < 0.05). The pain scores did not differ between groups. However, postoperative supplemental analgesia was required by significantly more cats in the Control (5/10) compared with the Laser group (1/10) (*P* = 0.038). Laser acupuncture reduced postoperative analgesic requirements in cats undergoing ovariohysterectomy.

## 1. Introduction

In recent decades, several studies have investigated the use of acupuncture for analgesic purposes and reported its effectiveness for the relief of both acute and chronic pain [1–5].

Traditionally, acupuncture is based on the philosophy of energy balance, so that any alteration, block, or stagnation in the flow of energy circulating through the body can promote the development of disease or pain [6]. From the point of view of traditional oriental medicine, pain is due to the stagnation of energy (Qi) and/or blood (Xue) flow along the meridians. Thus, the energy can be renewed and the body rebalanced by inserting needles at specific points, called acupoints [7, 8].

In addition to needles, stimulation of the acupuncture points can also be triggered through electrical stimulation [1–3], radiation (infrared laser) [5], and heat (moxibustion) [7].

Low intensity lasers can be used directly on the acupuncture points for treatment of pain [5, 9]. The main advantages of this technique over traditional acupuncture are shorter session length and the absence of discomfort and risk of infection, due to the noninvasive technique [10]. In rats subjected to experimental models of inflammation and pain, manual stimulation (needling) was as effective as laser stimulation at the same acupoint, resulting in an increased nociceptive threshold and reduced inflammation [11].

In human medicine, this therapeutic approach is common in paediatrics and in patients with needle phobias [10, 12]. In veterinary medicine, this technique may represent a viable alternative for those patients where introduction of a needle may be hindered by the behavior of the animal. In this context, cats represent a suitable population for the use of this mode of acupuncture, due to their quick temper and greater vulnerability to stress and irritability which can be triggered by needling acupoints [13].

Thus, the objective of this study was to evaluate the application of an infrared laser to acupuncture points as an adjuvant for postoperative pain management in cats undergoing ovariohysterectomy (OH).

## 2. Methods

This study was performed following the guidelines of the Brazilian College of Animal Experimentation, and the experimental procedure was approved by the Institutional Animal Care Committee (protocol 1975, CEEA). Informed consent was obtained from all of the cats' owners.

### 2.1. Animals

Twenty crossbreed cats, aged 6 to 36 months (27.3 ± 0.6 months) and weighing between 2.0 and 3.6 kg (median, 2.7 kg) undergoing OH, were enrolled. The cats were selected for this study after a physical examination and laboratory tests (i.e., complete blood cell count and measurements of urea, creatinine, alanine aminotransferase, and aspartate aminotransferase levels). The exclusion criteria were animals with alterations in blood count and/or liver and kidney functions.

### 2.2. Experimental Groups

**Figure 1:**
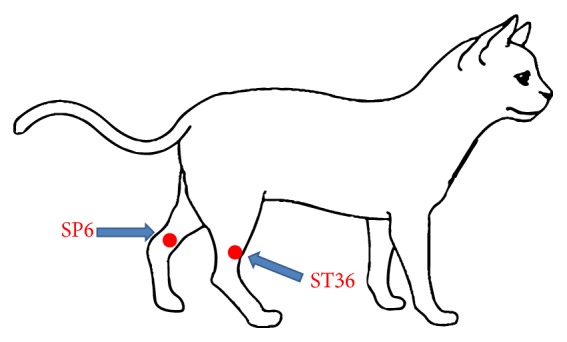
Location of ST36 and SP6 acupoints.

Prior to induction of general anaesthesia, the subjects were randomly distributed into two groups of 10 cats: Control (*n* = 10): no stimulation with acupuncture was performed; Laser (*n* = 10): there was application with an infrared laser (Gallium arsenate, AsGA, Laserpulse, Ibramed, Brazil) to the Zusanli (ST36) and Sanyinjiao (SP6) acupoints, bilaterally according to the following specifications: 904 nm wave length, 124 Hz, and 3 J/cm^2^ applied for 9 seconds to each acupoint [11]. The stimulation sequence was ST36 right, ST36 left, SP6 right, and SP6 left. The Zusanli (ST36) acupoint is located 3 cun (1 cun = width of the last rib) distal to the lateral head of the fibula. The acupoint Sanyinjiao (SP6) is located 3 cun proximal to the medial malleolus, at the caudal border of the tibia, close to the medial saphenous vein [15] (Figure 1). Cats in both groups had the skin over the acupoints shaved.

### 2.3. Surgical and Anesthetic Procedures

After fasting periods of 12 and 3 hours for solids and water, respectively, all animals were sedated intramuscularly with 5 mg kg^−1^ ketamine (Cetamin, Syntec, Brazil), associated with 0.5 mg kg^−1^ of midazolam (Dormonid, Cristália, Brazil) and 2 mg kg^−1^ of tramadol (Tramadon, Cristália, Brazil), in the same syringe. Fifteen minutes later the animals were placed in a secluded, quiet room, with no traffic of people and dim lighting, where the catheterization was performed (Insyte, Becton Dickinson, Brazil) in the cephalic vein, followed by the administration of Ringer's lactate solution (5 mL kg^−1^ h^−1^) by peristaltic infusion pump (Santronic, Brazil), which was maintained until the end of the surgical procedure. During this period, in addition to fluid treatment, acupuncture was performed in the Laser group. Anaesthesia was induced intravenously with propofol (Propovan, Cristália, Brazil), in dose-dependent effect. Immediately afterwards, orotracheal intubation was performed, and anaesthesia was maintained with isoflurane (Isoforine, Cristália, Brazil) in 100% oxygen at a rate of 400 mL kg^−1^ min^−1^ using a small animal circuit without reinhalation of gases (Baraka, Takaoka, Brazil). During the anaesthetic procedure, the end-tidal carbon dioxide concentration (ETCO_2_), end-tidal isoflurane concentration (ET_ISO_), oxygen saturation of hemoglobin (SpO_2_%), heart rate (HR), respiratory rate (RR), and esophageal temperature (T) were continuously measured using a multiparametric monitor (VAMOS plus; Dräger). Systolic arterial blood pressure (SBP) was measured using a noninvasive method with a Doppler ultrasonic system (Doppler 841-A; Parks Medical Electronics), with the width cuff approximately 40% of the antebrachium circumference. The end-tidal concentration of isoflurane (ET_ISO_) was adjusted based on arterial pressure and HH changes and using the conventional signs of anaesthesia [16].

The surgical procedures were performed by the same surgeon, who used the same surgical technique and suture material for all the animals.

### 2.4. Evaluation of the Degree of Analgesia

The animal pain score was evaluated preoperatively (24 h prior to surgery) and postoperatively (0.5, 1, 2, 4, 6, 8, 12, 18, and 24 h after tracheal extubation) using the Dynamic Interactive Visual Analogue Scale (DIVAS) [17, 18] and Multidimensional Composite Pain Scale (MCPS) [19].

For evaluation by DIVAS a 100 mm line was used, with the far left (=0) representing the animal with no signs of pain and the extreme right (=100), maximum pain. The DIVAS pain scoring involved three different phases. Initially, the cat was individually evaluated for 1 minute in its cage. Following this, the animal was stimulated to move around, in order for reactions and behaviour to be observed. Finally, the incision and surrounding area of the abdomen were palpated using 2-3 digits [18].

The MCPS was obtained on the basis of posture, comfort, activity, attitude, vocalization, and interactive behaviour, including palpation of the surgical wound, and abdomen/flank (see Appendix section). Each of the above-mentioned categories contained four descriptive behaviours (0 = normal unaffected behaviour and posture and 4 = severe changes). The observer chose a description in each category that best fits the cat's condition, so that the maximum score obtained was 24 points.

The administration of postoperative analgesic drugs was supervised by one anesthesiologist, and the pain assessment was performed by a blinded assessor. If the pain scores exceeded 33% of DIVAS and/or MCPS, 2 mg kg^−1^ tramadol (Tramadon, Cristália, Brazil) was administered intramuscularly as a rescue analgesic. Thirty minutes after the first supplemental analgesia, if the DIVAS and/or MCPS score remained above 33%, meloxicam was administered at a dose of 0.2 mg kg^−1^ (IM). The number of additional administrations of tramadol and/or meloxicam and the interval between them were recorded.

### 2.5. Statistical Analysis

The statistics were performed using analysis of variance (ANOVA) followed by Tukey's test to compare differences between the means of the different groups, for the parametric variables (HR, *f*, SBP, SpO_2_, ETCO_2_, ET_ISO_, surgical time, and time of extubation). The scores obtained from the evaluation of the degree of analgesia were evaluated using the Kruskal-Wallis test to compare the differences between the groups over time, while the Friedman test was used to compare the differences over time within each group. Dunn's posttest was used when significant differences were detected. The analyses were carried out on a standard PC microcomputer using the GraphPad Instat5 program with a significance level of 5%.

## 3. Results

There was no significant difference between treatments in relation to body weight, age, duration of surgery, and anaesthetic recovery time (Table 1).

The intraoperative HR (Control: 157 ± 19, 120–204 beats min^−1^; Laser: 165 ± 20, 119–222 beats min^−1^), SBP (Control: 145 ± 22, 117–150 mmHg; Laser: 134 ± 25, 100–170 mmHg), RR (Control: 24 ± 10, 10–30 breaths min^−1^; Laser: 22 ± 20, 10–28 breaths min^−1^), T (Control: 36.6 ± 1.2, 36–37.5°C; Laser: 36.6 ± 1.1, 36–38.1°C), ETCO_2_ (Control: 37 ± 0.5, 32–45 mmHg; Laser: 38 ± 2, 30–43 mmHg), SpO_2_ (Control: 99 ± 1, 97–100%; Laser: 98 ± 0.6, 97–99%), and ET_ISO_ (Control: 1.07 ± 0.25, 0.9–1.7%; Laser: 1.10 ± 0.10, 1.0–1.6%) concentrations were not significantly different between treatment groups at any time point (*P* > 0.05).

The median pain scores (DIVAS and MCPS) did not significantly differ between the treatment groups at any time point (*P* > 0.05). The pain scores were higher than the corresponding baseline values in the first 4 h after extubation in both treatment groups.

The need for supplemental postoperative analgesia was significantly lower (*P* = 0.038) in the Laser treatment group (one animal, one dose tramadol) compared to the Control group (5 animals, with a total of 5 doses of tramadol and 3 doses of meloxicam) (Table 2).

## 4. Discussion

This study showed that cats which received laser acupuncture had a significantly lower incidence of rescue medication than the Control group, suggesting a superior level of analgesia when acupuncture was administered preoperatively.

The analgesic effect mediated by acupuncture is closely related to the points stimulated. In the current study, the acupoints stimulated were selected based on previous reports confirming the analgesic potential of points ST36 and SP6 for the control of acute postoperative pain [1, 14, 20]. The combined stimulus of acupoints ST36 and SP6 can promote increased blood circulation and energy, in addition to triggering an anti-inflammatory and analgesic effect [21].

In addition to the choice of acupoints, other factors that can interfere with the analgesic response are the characteristics of the applied stimuli. In treatment with laser acupuncture, the use of an infrared laser with a wavelength between 650 and 1000 nm has a penetration depth of 2 to 3 mm [22], being capable of triggering a feeling of DeQi, which represents a determining factor for the effective stimulation of the acupoint [16, 23]. Additionally, the intensity of the applied radiation may interfere with the analgesic effect. Clinical studies have reported satisfactory analgesia after infrared laser treatment, using radiation intensities ranging from 3 to 10 J/cm^2^ [24, 25]. Thus, the radiation characteristics (wavelength 904 nm; intensity of 3 J/cm^2^) used in the present study may have contributed to the analgesic effect.

There is evidence that stimulation of acupuncture points is able to activate the descending inhibitory system in the spinal cord, brainstem, and other areas of the central nervous system, such as the thalamus, diencephalon, hypothalamus, and hypophysis [26–28]. Additionally, the release of endogenous opioids such as endorphins, enkephalins, and dynorphins also contributes to the analgesic effect mediated by acupuncture [28]. In addition to the opioid peptides, other factors are involved in the neurochemical mechanisms of acupuncture analgesia such as serotonin, norepinephrine, dopamine, acetylcholine, gamma-aminobutyric acid, substance P, glutamate, cyclic AMP, calcium ions, and endogenous cannabinoids [6, 29]. Erthal et al. [11] demonstrated the involvement of the opioidergic and serotonergic systems in the antinociceptive effect mediated by stimulation with laser of acupoint ST36 in rats. Therefore, it is suggested that treatment with laser acupuncture potentiated the analgesia mediated by tramadol, whose analgesic properties are attributed to opioid mechanisms and inhibiting the reuptake of serotonin and noradrenalin [30].

However, despite the fact that treatment with laser acupuncture have promoted a reduction in the consumption of analgesics in the postoperative period, pain scores did not differ between treatments. This result is probably associated with increased consumption of analgesics in the Control group, which allowed the reduction in pain scores and hence masked the differences between the groups. The need for supplemental analgesia was observed during the first 4 hours after surgery, being implemented in 10% and 50% of animals in the Laser and Control groups, respectively. In a similar study the need for postoperative analgesic supplementation was reported in 50% of the cats submitted to OH, treated with tramadol in the preoperative period [17].

The assessment of pain in cats is difficult due to the impossibility of verbal communication between the evaluator and the patient. The DIVAS is an interval scale, regularly used by the scientific community to assess pain in cats [17, 18]. Additionally, the MCPS is a valid, reliable, and responsive scale for the assessment of acute pain in cats [19, 31]. Therefore, we sought to employ the most appropriate pain evaluation scales in order to minimize potential interference with the recognition of discomfort in the animal. In addition, all animals were treated by the same surgeon and pain measurement was performed by the same observer, who did not know to which treatment the animal belonged.

One of the limiting factors of the current study was the lack of a control group without treatment with analgesics in the preoperative period. It is possible that the inclusion of this group would have enabled the detection of statistically significant differences in pain scores, promoting greater consistency in the results obtained. However, because the cats in the study originated from a hospital routine and for ethical reasons, we chose not to include this group, since previous studies have shown that postoperative pain was detected in 100% of cats submitted to OSH without preventive analgesic treatment, requiring supplemental analgesia [19, 31].

In summary, our results demonstrate that laser acupuncture reduces postoperative analgesic requirements in cats undergoing ovariohysterectomy.

## Figures and Tables

**Table 1:** Mean ± standard deviations of the body weight, age, and surgical and anaesthetic recovery times of cats undergoing ovariohysterectomy treated with laser acupuncture (Laser, *n* = 10) or no acupuncture treatment (Control, *n* = 10).

	Body weight (kg)	Age (months)	Surgery time (min)	Extubation time (min)	Recovery time (min)
Laser	2.6 ± 0.6	26 ± 16	11 ± 4	7 ± 5	39 ± 7
Control	2.8 ± 0.4	28 ± 18	12 ± 3	6 ± 2	36 ± 9

**Table 2:** Number of rescue doses administered over time in cats undergoing ovariohysterectomy treated with laser acupuncture (Laser, *n* = 10) or no acupuncture treatment (Control, *n* = 10).

	Groups	Postoperative time (h)								Total
		0.5	1	2	4	8	12	18	24	
Rescue doses (number)	Control	1	3	2	2					8^#^
	Laser	1								1

^#^Significantly different from Control group (Mann-Whitney *U* test, *P* = 0.038).

**Table 3:** Multidimensional Composite Pain Scale (Brondani et al., 2011 [31]).

Posture	The cat is in a natural posture with relaxed muscles (it moves normally)	0
	The cat is in a natural posture but is tense (it moves little or is reluctant to move)	1
	The cat is sitting or is in sternal recumbency with its back arched and head down; or the cat is in dorsolateral recumbency with its pelvic limbs extended or contracted	2
	The cat frequently alters its body position in an attempt to find a comfortable posture	3

Comfort	The cat is comfortable, awake, or asleep and interacts when stimulated (it interacts with the observer and/or is interested in its surroundings)	0
	The cat is quiet and slightly receptive when stimulated (it interacts little with the observer and/or is not very interested in its surroundings)	1
	The cat is quiet and is “dissociated from the environment” (even when stimulated it does not interact with the observer and/or has no interest in its surroundings); the cat may be facing the back of the cage	2
	The cat is uncomfortable, restless (frequently changes its body position), and slightly receptive when stimulated or “dissociated from the environment”; the cat may be facing the back of the cage	3

Activity	The cat moves normally (it immediately moves when the cage is opened; outside the cage it moves spontaneously when stimulated or handled)	0
	The cat moves more than normal (inside the cage it moves continuously from side to side)	1
	The cat is quieter than normal (it may hesitate to leave the cage and if removed from the cage tends to return; outside the cage it moves a little after stimulation or handling)	2
	The cat is reluctant to move (it may hesitate to leave the cage and if removed from the cage tends to return; outside the cage it does not move even when stimulated or handled)	3

Attitude	A: satisfied, the cat is alert, is interested in its surroundings (explores its surroundings), and is friendly and interactive with the observer (plays and/or responds to stimuli); the cat may initially interact with the observer through games to distract it from the pain; carefully observe to distinguish between distraction and satisfaction games; B: uninterested, the cat does not interact with the observer (is not interested in toys or plays a little and does not respond to calls or strokes from the observer); in cats which do not like to play, evaluate interaction with the observer by its response to calls and strokes; C: indifferent, the cat is not interested in its surroundings (it is not curious; it does not explore its surroundings); the cat can initially be afraid to explore its surroundings; the observer needs to handle the cat and encourage it to move itself (take it out of the cage and/or change its body position); D: anxious, the cat is frightened (it tries to hide or escape) or nervous (demonstrating impatience and growling, howling, or hissing when stroked and/or handled); E: aggressive, the cat is aggressive (tries to bite or scratch when stroked or handled)	
	Presence of the mental state A	0
	Presence of one of the mental states B, C, D, or E	1
	Presence of two of the mental states B, C, D, or E	2
	Presence of three or all of the mental states B, C, D, or E	3

Miscellaneous behaviors	A: the cat is lying down and is quiet but is moving its tail. B: the cat contracts and extends its pelvic limbs and/or contracts its abdominal muscles (flank); C: the cats eyes are partially closed (eyes half closed); D: the cat licks and/or bites the surgical wound	
	All of the above behaviors are absent	0
	Presence of one of the above behaviors	1
	Presence of two of the above behaviors	2
	Presence of three or all of the above behaviors	3

Reaction to palpation of the surgical wound	The cat does not react when the surgical wound is touched or pressed; or there is no change from presurgical response (if basal evaluation was made)	0
	The cat does not react when the surgical wound is touched but does react when it is pressed; it may vocalize and/or try to bite	1
	The cat reacts when the surgical wound is touched and when it is pressed; it may vocalize and/or try to bite	2
	The cat reacts when the observer approaches the surgical wound; it may vocalize and/or try to bite; the cat does not allow palpation of the surgical wound	3

Reaction to palpation of the abdomen/flank	The cat does not react when the abdomen/flank is touched or pressed; or there is no change from presurgical response (if basal evaluation was made); the abdomen/flank is not tense	0
	The cat does not react when the abdomen/flank is touched but does react when it is pressed; the abdomen/flank is tense	1
	The cat reacts when the abdomen/flank is touched and when it is pressed; the abdomen/flank is tense	2
	The cat reacts when the observer approaches the abdomen/flank; it may vocalize and/or try to bite; the cat does not allow palpation of the abdomen/flank	3

Vocalization	The cat is quiet, is purring when stimulated, or miaows interacting with the observer but does not growl, groan, or hiss	0
	The cat purrs spontaneously (without being stimulated or handled by the observer)	1
	The cat growls, howls, or hisses when handled by the observer (when its body position is changed by the observer)	2
	The cat growls, howls, or hisses spontaneously (without being stimulated or handled by the observer)	3

## Appendix

See Table 3.
